# Efficacy and tolerability of lisdexamfetamine dimesylate in children with attention-deficit/hyperactivity disorder: sex and age effects and effect size across the day

**DOI:** 10.1186/1753-2000-4-32

**Published:** 2010-12-14

**Authors:** Sharon B Wigal, Scott H Kollins, Ann C Childress, Ben Adeyi

**Affiliations:** 1 University of California, Irvine, Child Development Center, Irvine, California, USA; 2 Duke University Medical Center, Durham, North Carolina, USA; 3 Center for Psychiatry and Behavioral Medicine, Las Vegas, Nevada, USA; 4 Shire Development Inc., Wayne, Pennsylvania, USA

## Abstract

**Background:**

Efficacy and safety profiles by sex and age (6-9 vs 10-12 years) and magnitude and duration of effect by effect size overall and across the day of lisdexamfetamine dimesylate (LDX) vs placebo were assessed.

**Methods:**

This study enrolled children (6-12 years) with attention-deficit/hyperactivity disorder (ADHD) in an open-label dose optimization with LDX (30-70 mg/d) followed by a randomized, double-blind, placebo-controlled, 2-way crossover phase. Post hoc analyses assessed interaction between sex or age and treatment and assessed effect sizes for Swanson, Kotkin, Agler, M-Flynn, and Pelham (SKAMP) and Permanent Product Measure of Performance (PERMP) scales and ADHD Rating Scale IV measures. No corrections for multiple testing were applied on time points and subgroup statistical comparisons.

**Results:**

129 participants enrolled; 117 randomized. Both sexes showed improvement on all assessments at postdose time points; females showed less impairment than males for SKAMP and PERMP scores in treatment and placebo groups at nearly all times. Both age groups improved on all assessments at postdose time points. Children 10-12 years had less impairment in SKAMP ratings than those 6-9 years. Treatment-by-sex interactions were observed at time points for SKAMP-D, SKAMP total, and PERMP scores; no consistent pattern across scales or time points was observed. LDX demonstrated significant improvement vs placebo, by effect size, on SKAMP-D from 1.5-13 hours postdose. The overall LS mean (SE) SKAMP-D effect size was -1.73 (0.18). In the dose-optimization phase, common (≥2%) treatment-emergent adverse events (TEAEs) in males were upper abdominal pain, headache, affect lability, initial insomnia, and insomnia; in females were nausea and decreased weight. During the crossover phase for those taking LDX, higher incidence (≥2% greater) was observed in males for upper abdominal pain and insomnia and in females for nausea and headache. Overall incidence of TEAEs in age groups was similar.

**Conclusion:**

Apparent differences in impairment level between sex and age groups were noted. However, these results support the efficacy of LDX from 1.5 hours to 13 hours postdose in boys and girls with medium to large effect sizes across the day with some variability in TEAE incidence by sex.

**Trial Registration Number:**

ClinicalTrials.gov Identifier: NCT00500149.

## Background

The efficacy and safety of stimulants for the pharmacologic management of attention-deficit/hyperactivity disorder (ADHD) is well documented [1,2]. Short-acting agents for the treatment of ADHD require multiple daily doses and have the potential for uneven symptom control [3,4]. After-school activities including sports or homework may last into later hours of the day, thus creating a need for long-acting stimulants for symptom control [3,5]. To address this and other limitations, novel delivery systems that result in longer durations of symptom control were developed [4-6].

Lisdexamfetamine dimesylate (LDX; Vyvanse^®^, Shire US Inc.) is a prodrug stimulant indicated for the treatment of ADHD in children (aged 6 to 12 years), adolescents (aged 13 to 17 years), and in adults. LDX is a therapeutically inactive molecule. After oral ingestion, LDX is converted to l-lysine and active d-amphetamine, which is responsible for the therapeutic effect [7]. In a 4-week, randomized, placebo-controlled, forced-dose titration trial in children with ADHD, LDX was administered in the morning with a median time of dose administration between 7:30 AM and 8:00 AM. LDX demonstrated efficacy versus placebo in improving ADHD symptoms by symptom ratings and global assessments from the first week of treatment through the end of the study [8]. In that study, LDX was well tolerated with a safety profile consistent with that of long-acting stimulant use. The most common adverse events (AEs) associated with LDX included decreased appetite, insomnia, abdominal pain, and irritability [8].

The onset and duration of efficacy of LDX in children was initially evaluated beginning 1 hour postdose and ending 12 hours postdose with significant efficacy shown from 2 to 12 hours [9]. A subsequent laboratory school study in children with ADHD evaluated onset and duration of efficacy from 1.5 to 13 hours postdose as measured by Swanson, Kotkin, Agler, M-Flynn, and Pelham (SKAMP) total and subscale scores. These results have been published elsewhere [10]. AEs in this study were consistent with those observed in other pediatric studies of LDX [8,9] with the exception of a higher-than-typically-seen increase in pulse at 12.5 hours postdose for the participants receiving 70 mg/d LDX [10].

LDX has been shown to be generally effective for treating ADHD symptoms across the day. Despite this, little is known about the moderating effects of age and sex on treatment response to LDX or other stimulants, and existing studies report mixed results [11]. Results from the Multimodal Treatment Study of Children With ADHD (MTA), a large community-based trial of children 7.0 to 9.9 years of age, indicated an overall lack of a moderating effect of sex on treatment response [12,13]. However, an analysis of data from the Comparison of Methylphenidates in an Analog Classroom Setting (COMACS) study, a classroom analog study of children 6 to 12 years of age, found that females had a stronger response to methylphenidate from 1.5 to 3.0 hours postdose; from 4.5 to 6.0 hours, responses in males and females were equivalent, whereas from 7.5 to 12 hours, response among females declined more quickly than among males, leading to better response in males for those time points [14]. The COMACS study included 2 active treatment arms, osmotic-release oral methylphenidate (OROS-MPH) and methylphenidate extended-release (MPH-ER). Interestingly, although differences existed in the efficacy profiles of OROS-MPH and MPH-ER in the overall group with MPH-ER demonstrating superiority early in the day and OROS-MPH demonstrating superiority later in the day [6], the differences between males and females were independent of formulation [14].

Although some differential cognitive functioning has been found between girls and boys, most studies have documented little difference between sexes in cognitive and executive functions while clearly documenting significant impairment in these domains in both girls and boys with ADHD compared to children without ADHD [15]. Depression and anxiety may be more problematic in girls than in boys [16], whereas boys with ADHD are consistently reported to be more disruptive, more commonly involved in rule breaking, and more likely to have comorbid disruptive behavior disorders [17,18].

Although the symptoms of ADHD may differ in patients of various age groups, little is known about differences in treatment effects and AEs experienced by children of different ages within the same study. In a study of preschool-aged children (3 to 5.5 years of age), treated with immediate-release methylphenidate in a 70-week, multiple phase study utilizing both parent and teacher-rated assessments (the Preschool ADHD Treatment Study [PATS]), factor scores derived from the Conners, Loney, and Milich scale observed smaller effect sizes in parent and teacher ratings (0.35 and 0.43, respectively) than those observed in results from the MTA study of school-aged children (0.52 and 0.75, respectively) [19,20]. In addition, the preschoolers in the PATS showed a higher rate of methylphenidate discontinuation due to spontaneously reported AEs than did school-aged children in the MTA study (ie, 11% vs <1%) [21]. In fact, pharmacokinetic differences seem to corroborate other variables such as clearance rate contributing to age-related differences [22]. Although many factors may have contributed to these differences, they raise the question of whether certain age groups benefit more from treatment than do others.

Dosing of stimulants is not generally based on by-weight guidelines, and younger, smaller children may receive relatively higher by-weight doses than do older, larger children. Younger, smaller children may theoretically be at higher risk for dose-dependent AEs. This possibility is supported by findings that these participants were prone to higher incidence of some AEs when receiving the highest dose level in a study of long-acting methylphenidate [23]. Participants in the PATS experienced loss of appetite, trouble sleeping, stomachaches, social withdrawal, and lethargy, and these AEs occurred more frequently in participants receiving high-dose methylphenidate than in those receiving low-dose methylphenidate or placebo [19]. Younger participants may be more likely to experience sleep difficulties and decreased appetite as AEs with stimulant treatment at high dose levels [23].

The post hoc analyses presented here assessed the efficacy of LDX in female and male participants and in younger (6 to 9 years) and older (10 to 12 years) participants to determine whether sex or age interactions were present. The safety profile of LDX was further characterized by examining AEs by sex and age. Also assessed was the duration of efficacy of LDX in a laboratory school setting based on effect size calculations for SKAMP and Permanent Product Measure of Performance (PERMP) measures. Effect size analyses are a useful method for providing clinically relevant information about the magnitude of effect relative to the effects of placebo, and where data are available, effect size assessments provide a systematic quantitative framework for assessing the relative effects of therapeutic agents across studies [24]. Effect size analyses may provide more practical information about the expected therapeutic effect (eg, efficacy and tolerability) that can be applied to making therapeutic choices.

### Study objectives

The objective of this post hoc analysis was to examine the efficacy and safety profile of LDX by sex and age group in children with ADHD in a laboratory school setting. This analysis also aimed to assess the magnitude of effect overall and across the day of LDX vs placebo based on effect size analysis of SKAMP, PERMP, and ADHD Rating Scale IV (ADHD-RS-IV) scores.

## Methods

**Figure 1:**
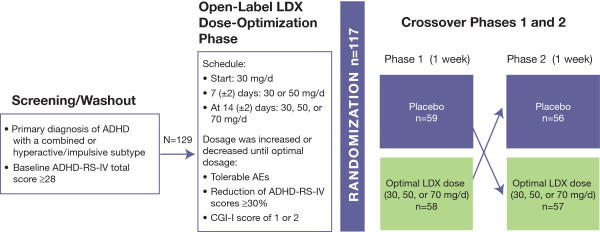
**Study design**. Twelve participants discontinued prior to randomization; 4 participants discontinued during the crossover period; 2 participants discontinued after the crossover phase; 9 participants discontinued because of AEs; no participants discontinued because of lack of efficacy.

This randomized, double-blind, multicenter, placebo-controlled, dose-optimization, crossover laboratory school study of LDX was conducted at 7 study sites in the United States. Full details of the methodological design and conduct of this study have been previously published [10]. All study activities were performed in accordance with the principles of the International Conference on Harmonisation Good Clinical Practice, 18th World Medical Assembly (Helsinki 1964), and amendments of the 29th (Tokyo 1975), the 35th (Venice 1983), the 41st (Hong Kong 1989), and the 48th (South Africa 1996) World Medical Assemblies. This was a study of children (6 to 12 years of age) diagnosed with moderate to severe ADHD (baseline ADHD-RS-IV score ≥28) and included a screening and washout (for those participants taking other medications for ADHD at screening), an open-label, dose-optimization phase of LDX (30, 50, or 70 mg/d) followed by a randomized, double-blind, placebo-controlled, 2-way crossover phase (Figure 1). Key exclusion criteria were the presence of a comorbid psychiatric condition with severe symptoms, conduct disorder, or other medical condition that could confound assessments, pose a risk to the participant, or prohibit study completion. Other inclusion and exclusion criteria were detailed previously [10].

### Efficacy measures

Efficacy evaluations were performed on the intention-to-treat population (ITT) population, defined as all participants who were randomized and had at least 1 SKAMP-Deportment (SKAMP-D) score available after randomization. Efficacy measures were collected in the analog classroom setting at predose (-0.5 hours) and 1.5, 2.5, 5.0, 7.5, 10.0, 12.0, and 13.0 hours postdose during crossover periods 1 and 2. The primary efficacy outcome was the onset of efficacy for LDX vs placebo as assessed by the primary outcome measure, SKAMP-D scores [10]. Key secondary assessments included the SKAMP-Attention (SKAMP-A) and SKAMP quality of work subscales, SKAMP total scores, PERMP number attempted (PERMP-A) and PERMP number correct (PERMP-C), and the ADHD-RS-IV. SKAMP and PERMP were assessed predose and 1.5, 2.5, 5.0, 7.5, 10.0, 12.0, and 13.0 hours postdose during crossover periods 1 and 2. ADHD-RS-IV was administered at baseline and during each weekly visit.

The SKAMP scale is a validated rating scale that assesses behavioral symptoms of ADHD in a classroom setting using a 7-point impairment scale (0 = none, 6 = maximal impairment) [25,26]. The SKAMP total score comprises 13 items [26]. The SKAMP-D subscale evaluates deportment, including interacting with other children, interacting with adults, remaining quiet according to classroom rules, and staying seated according to classroom rules. The SKAMP-A subscale is a measure of attention and evaluates getting started on assignments, sticking with tasks, attending to an activity, and making activity transitions. The SKAMP quality of work subscale includes 3 items: completing assigned work, performing work accurately, and being careful and neat while writing or drawing.

The PERMP, a 5-page test consisting of 80 math problems per page (total of 400 problems) [26], evaluated effortful performance in the classroom as a measure of efficacy. Participants were instructed to work at their seats and to complete as many problems as possible in 10 minutes. The appropriate level of difficulty for each student was determined previously based on results of a math pretest administered at screening. Performance was evaluated using PERMP-A and PERMP-C scores.

The ADHD-RS-IV [27] is a clinician-rated scale that reflects current symptoms of ADHD based on *Diagnostic and Statistical Manual of Mental Disorders, Fourth Edition, Text Revision *(DSM-IV-TR) criteria; it is a global assessment that measures the severity of symptoms from visit to visit, but was not used to assess symptoms of ADHD over the course of the day. The ADHD-RS-IV consists of 18 items that are grouped into 2 subscales (hyperactivity/impulsivity and inattention). Each item is scored on a scale of 0 (no symptoms) to 3 (severe symptoms), yielding a total score of 0 to 54.

### Safety assessments

The safety population included all participants who were enrolled in the dose-optimization phase and received at least 1 dose of LDX. Treatment-emergent AEs (TEAEs), referring to events with onset after the first date of treatment, and no later than 3 days following termination of treatment, were recorded separately for the dose-optimization and the double-blind crossover phases of the study. TEAEs that continued uninterrupted from the dose-optimization to the crossover phase without a change in severity were counted only in the dose-optimization phase category. TEAEs with a change in severity across phases or that resolved and then restarted in the crossover phase were counted both in the dose-optimization and crossover arms. TEAEs for which a missing or incomplete start date made it impossible to determine in which phase of the study they started were counted as starting in the dose-optimization phase. TEAEs were reported as number and percentage of participants according to system-organ class, preferred term, treatment group, and by last dose received at AE onset. AEs were collected at all visits by soliciting participant report with nonleading questions, and were coded using the *Medical Dictionary for Regulatory Activities *(MedDRA).

### Statistical analyses

Treatment interaction by age and sex were analyzed, post hoc, among the ITT population using a linear mixed model with sequence, period, sex (or age), treatment, and treatment by sex (or age) defined as fixed effects and subject-within-sequence as the random effect. No corrections for multiple testing were applied on time points and subgroup statistical comparisons.

Post hoc analyses evaluated SKAMP and PERMP effect size calculations for different dose groups, SKAMP and PERMP scores for males and females, and demographic data and AEs by age and sex. Least squares (LS) effect size and standard errors (SEs) were calculated according to the method of Curtin, Altman, and Elbourne [28] (standardized weighted mean difference [SWMD] methodology) at each postdose time point for SKAMP and PERMP assessments and for mean SKAMP-D score in the ITT population. The SWMD considers the treatment effect in relation to within-group standard deviation (SD) to combine continuous results of trials to evaluate the effect of treatment [28]. Effect size is a derived statistical assessment designed to allow comparisons of efficacy across clinical trials [29]. In general, effect size is calculated as the difference between drug effects and placebo effects divided by their pooled SD [29]. There are multiple methods for assessing pooled variance [28] depending on differences in the study design of included studies. Based on analysis by Cohen, effect sizes of 0.2, 0.5, and 0.8, respectively, correspond to a small, medium, and large magnitude of effect [30]. Negative SKAMP effect sizes and positive PERMP effect sizes indicate improvement with LDX.

## Results

During the dose-optimization phase, 58 participants were optimized to 30 mg/d LDX, 50 participants to 50 mg/d LDX, and 21 participants to 70 mg/d LDX; across all groups, participant demographics and characteristics were well balanced (Table 1). Overall demographic data have been published previously [10].

**Table 1:** Participant Demographics (Safety Population)

				LDX Dose			
				
Category		Age Group	Statistic	30 mg/d	50 mg/d	70 mg/d	All doses
Age (y)		6-9	n	27	15	6	48
			Mean (SD)	8.5 (0.75)	8.5 (0.74)	8.0 (0.89)	8.5 (0.77)
	
		10-12	n	31	35	15	81
			Mean (SD)	10.9 (0.96)	10.9 (0.73)	11.4 (1.12)	11.0 (0.91)

Sex*	Male	6-9	n (%)	18 (66.7)	13 (86.7)	6 (100)	37 (77.1)
	Female		n (%)	9 (33.3)	2 (13.3)	0	11 (22.9)
	
	Male	10-12	n (%)	26 (83.9)	24 (68.6)	11 (73.3)	61 (75.3)
	Female		n (%)	5 (16.1)	11 (31.4)	4 (26.7)	20 (24.7)

Weight (lb)		6-9	n	27	15	6	48
			Mean (SD)	62.2 (8.78)	65.0 (13.75)	53.1 (2.50)	61.9 (10.62)
	
		10-12	n	31	35	15	81
			Mean (SD)	78.9 (16.36)	79.0 (18.64)	80.7 (17.29)	79.3 (17.34)

Height (in)		6-9	n	27	15	6	48
			Mean (SD)	51.2 (2.30)	52.1 (2.29)	49.3 (2.04)	51.3 (2.38)
	
		10-12	n	31	35	15	81
			Mean (SD)	56.2 (2.84)	56.2 (3.08)	57.3 (2.79)	56.4 (2.93)

Body mass index (kg/m^2^)		6-9	n	27	15	6	48
			Mean (SD)	16.6 (1.44)	16.7 (2.38)	15.4 (1.28)	16.5 (1.78)
	
		10-12	n	31	35	15	81
			Mean (SD)	17.4 (2.45)	17.4 (2.67)	17.2 (2.55)	17.4 (2.53)

SD: standard deviation.

*Percentages are based on number of participants in the age group of the dose classification.

In the 2-way crossover phase of the study, 129 participants were enrolled and 117 were randomized. Eighteen participants discontinued the study with 9 discontinuing because of AEs. No participant discontinued because of lack of efficacy of LDX (Figure 1).

### Efficacy analyses

#### Sex analysis

At the predose time point, there were significant effects of sex for SKAMP-D and SKAMP-A subscale scores and SKAMP total scores and significant treatment condition effects for SKAMP-A and SKAMP quality of work subscale scores and SKAMP total scores (Table 2). There were significant treatment condition effects for PERMP-A and PERMP-C, and no significant effects of treatment-by-sex interactions were observed at the predose time point (Table 2).

**Table 2:** Mixed Model Analysis by Treatment, Sex, and Treatment by Sex for Predose and Postdose Time Points*

Time Point (hr)	Mixed Model Statistical Analysis	SKAMP-D		SKAMP-A		SKAMP-Total		SKAMP-QoL		PERMP-A		PERMP-C	
		**F Value**	***P *Value**	**F Value**	***P *Value**	**F Value**	***P *Value**	**F Value**	***P *Value**	**F Value**	***P *Value**	**F Value**	***P *Value**

**-0.5**	**Treatment**	0.99	.3230	7.21	.0084	28.52	<.0001	93.12	<.0001	22.19	<.0001	23.58	<.0001
	
	**Sex**	**9.36**	**.0028**	**8.71**	**.0039**	**13.38**	**.0004**	**0.75**	**.3891**	**0.31**	**.5809**	**0.25**	**.6164**
	
	**Treatment by sex**	1.95	.1657	0.19	.6620	2.49	.1173	0.52	.4714	0.59	.4447	0.76	.3861

**1.5**	**Treatment**	**19.58**	**<.0001**	**12.45**	**.0006**	**28.01**	**<.0001**	**1.06**	**.3049**	**13.67**	**.0003**	**19.73**	**<.0001**
	
	**Sex**	6.59	.0116	3.50	.0639	7.84	.0060	3.72	.0562	0.37	.5421	0.48	.4899
	
	**Treatment by sex**	**0.00**	**.9779**	**0.10**	**.7528**	**0.20**	**.6578**	**0.88**	**.3493**	**0.05**	**.8245**	**0.39**	**.5315**

**2.5**	**Treatment**	70.77	<.0001	53.15	<.0001	131.44	<.0001	43.18	<.0001	60.11	<.0001	65.68	<.0001
	
	**Sex**	**7.89**	**.0059**	**2.35**	**.1278**	**8.72**	**.0038**	**2.88**	**.0928**	**0.39**	**.5326**	**0.39**	**.5350**
	
	**Treatment by sex**	2.71	.1023	0.81	.3708	2.97	.0875	0.15	.6995	1.10	.2975	1.62	.2063

**5.0**	**Treatment**	**74.56**	**<.0001**	**50.75**	**<.0001**	**136.60**	**<.0001**	**68.23**	**<.0001**	**73.30**	**<.0001**	**76.40**	**<.0001**
	
	**Sex**	7.13	.0087	2.58	.1111	7.00	.0093	0.20	.6518	0.23	.6357	0.18	.6708
	
	**Treatment by sex**	**2.50**	**.1166**	**1.33**	**.2517**	**1.63**	**.2049**	**0.28**	**.5949**	**1.18**	**.2801**	**1.40**	**.2398**

**7.5**	**Treatment**	67.32	<.0001	56.67	<.0001	137.48	<.0001	53.75	<.0001	89.27	<.0001	92.70	<.0001
	
	**Sex**	**9.38**	**.0028**	**3.52**	**.0633**	**11.68**	**.0009**	**10.46**	**.0016**	**1.65**	**.2021**	**1.73**	**.1916**
	
	**Treatment by sex**	4.31	.0402	2.10	.1506	4.16	.0438	0.24	.6248	0.92	.3406	0.76	.3855

**10**	**Treatment**	**44.74**	**<.0001**	**39.70**	**<.0001**	**92.89**	**<.0001**	**17.42**	**<.0001**	**64.41**	**<.0001**	**69.95**	**<.0001**
	
	**Sex**	8.67	.0039	5.07	.0263	13.35	.0004	3.22	.0754	0.65	.4233	0.69	.4083
	
	**Treatment by sex**	**1.51**	**.2222**	**3.67**	**.0579**	**6.31**	**.0135**	**2.86**	**.0939**	**5.43**	**.0217**	**4.98**	**.0277**

**12**	**Treatment**	21.05	<.0001	30.04	<.0001	69.72	<.0001	26.38	<.0001	47.55	<.0001	52.36	<.0001
	
	**Sex**	**14.36**	**.0002**	**2.33**	**.1299**	**12.84**	**.0005**	**0.67**	**.4165**	**0.34**	**.5613**	**0.36**	**.5484**
	
	**Treatment by sex**	0.93	.3374	0.37	.5455	0.55	.4586	0.31	.5767	1.32	.2524	1.42	.2357

**13**	**Treatment**	**3.70**	**.0568**	**19.09**	**<.0001**	**21.25**	**<.0001**	**8.13**	**.0052**	**39.39**	**<.0001**	**41.15**	**<.0001**
	
	**Sex**	12.45	.0006	2.84	.0945	13.56	.0004	1.53	.2187	1.08	.3012	1.37	.2439
	
	**Treatment by sex**	**1.30**	**.2571**	**2.69**	**.1039**	**3.03**	**.0844**	**0.00**	**.9942**	**3.58**	**.0609**	**2.74**	**.1006**

LDX: lisdexamfetamine dimesylate; PERMP: Permanent Product Measure of Performance; PERMP-A: PERMP-Attempted; PERMP-C: PERMP-Correct; SKAMP: Swanson, Kotkin, Agler, M-Flynn, and Pelham; SKAMP-A: SKAMP-Attention; SKAMP-D: SKAMP-Deportment.

*Degrees of freedom (df) = 110 for all analyses except the 7.5-hour postdose time point where df = 109 for all analyses.

**Figure 2:**
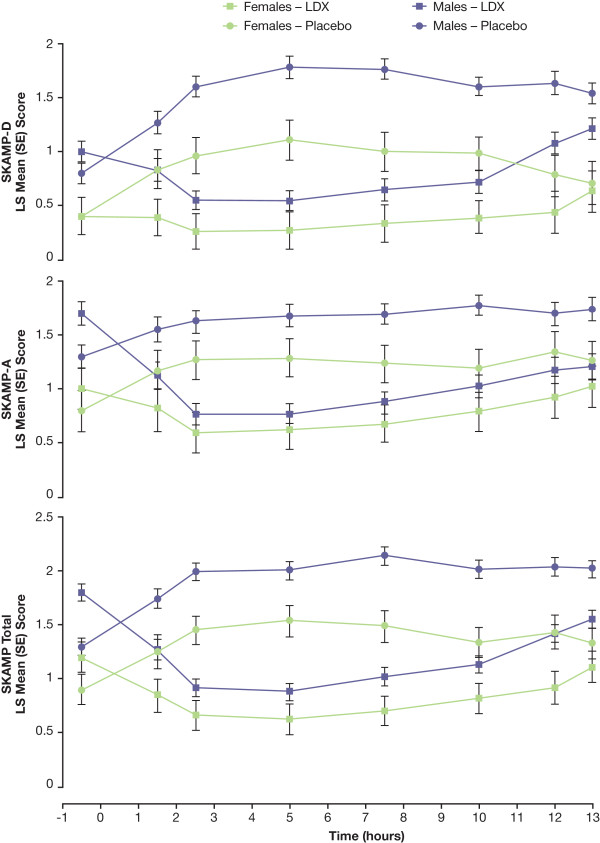
**Postdose LS Mean (SE) SKAMP-D, SKAMP-A, and Total Scores by Time and Sex**. Lower SKAMP scores indicate improvement.

**Figure 3:**
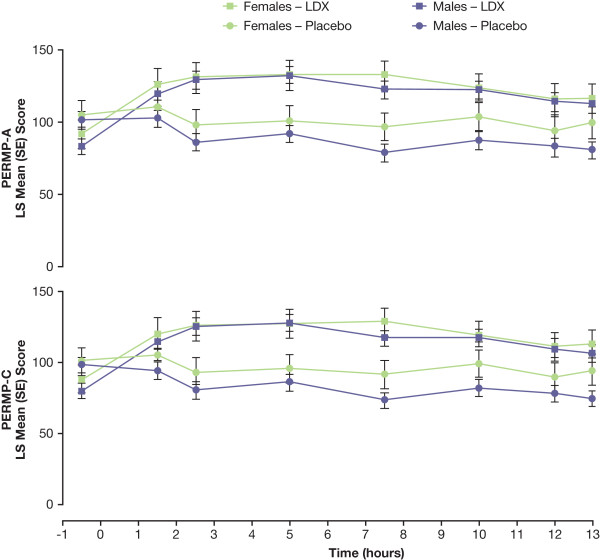
**Postdose LS Mean (SE) PERMP-A and PERMP-C Scores by Time and Sex**. Higher PERMP subscale scores are indicative of improvement.

Results of efficacy analyses for postdose time points by sex are shown in Figures 2 and 3 and mixed model statistical analysis in Table 2. There were significant effects of sex (*P *< .05) at all time points for SKAMP-D scores. For SKAMP-D scores, the only significant treatment-by-sex interaction was seen at the 7.5-hour time point. Results of the sex analysis for SKAMP total mirrored those of SKAMP-D with significant effects at all time points and a significant treatment-by-sex interaction at 7.5 and 10 hours postdose. For SKAMP-A, significant effects of sex were seen at only 1 time point (10 hours), and no significant treatment-by-sex interactions were observed at any postdose time point. For PERMP-A and PERMP-C, no significant effects of sex were seen, although significant treatment-by-sex interactions were seen at 1 time point (10 hours postdose). With LDX treatment, LS mean SKAMP scores for females were lower than those for males for all measures at all time points. Similarly, LS mean SKAMP scores for females were lower than those for males for all measures at all time points when receiving placebo.

#### Age analysis

At the predose time point, there were significant effects of age for SKAMP-D subscale and SKAMP total scores and significant treatment condition effects for SKAMP-A subscale, SKAMP quality of work subscale, SKAMP total scores, PERMP-A, and PERMP-C (Table 3).

**Table 3:** Mixed Model Analysis by Treatment, Age, and Treatment by Age for Predose and Postdose Time Points*

Time Point (hr)	Mixed Model Statistical Analysis	SKAMP-D		SKAMP-A		SKAMP-Total		SKAMP-QoL		PERMP-A		PERMP-C	
		**F Value**	***P *Value**	**F Value**	***P *Value**	**F Value**	***P *Value**	**F Value**	***P *Value**	**F Value**	***P *Value**	**F Value**	***P *Value**

**-0.5**	**Treatment**	3.69	.0574	10.88	.0013	48.67	<.0001	133.44	<.0001	34.98	<.0001	37.63	<.0001
	
	**Age**	**8.65**	**.0040**	**3.55**	**.0623**	**5.76**	**.0181**	**0.95**	**.3307**	**1.49**	**.2251**	**1.60**	**.2080**
	
	**Treatment by age**	0.10	.7574	0.72	.3971	0.31	.5771	0.02	.9027	0.20	.6580	0.17	.6793

**1.5**	**Treatment**	**27.82**	**<.0001**	**19.41**	**<.0001**	**42.98**	**<.0001**	**2.92**	**.0903**	**18.95**	**<.0001**	**30.00**	**<.0001**
	
	**Age**	6.83	.0102	6.63	.0113	6.93	.0097	0.03	.8716	0.59	.4430	0.54	.4627
	
	**Treatment by age**	**2.38**	**.1255**	**1.91**	**.1693**	**2.32**	**.1307**	**0.09**	**.7625**	**0.49**	**.4876**	**0.02**	**.8854**

**2.5**	**Treatment**	121.66	<.0001	79.31	<.0001	204.35	<.0001	61.19	<.0001	94.10	<.0001	103.60	<.0001
	
	**Age**	**7.25**	**.0082**	**3.70**	**.0571**	**7.99**	**.0056**	**2.49**	**.1172**	**0.14**	**.7066**	**0.23**	**.6303**
	
	**Treatment by age**	5.99	.0159	0.01	.9253	1.93	.1672	0.02	.8971	1.74	.1902	1.27	.2619

**5.0**	**Treatment**	**126.22**	**<.0001**	**79.64**	**<.0001**	**213.18**	**<.0001**	**89.58**	**<.0001**	**117.59**	**<.0001**	**122.97**	**<.0001**
	
	**Age**	5.20	.0245	6.59	.0116	8.64	.0040	2.49	.1173	0.04	.8480	0.07	.7974
	
	**Treatment by age**	**5.57**	**.0201**	**0.49**	**.4865**	**4.54**	**.0353**	**2.72**	**.1018**	**4.41**	**.0379**	**4.25**	**.0415**

**7.5**	**Treatment**	109.78	<.0001	98.23	<.0001	215.28	<.0001	76.54	<.0001	134.00	<.0001	137.53	<.0001
	
	**Age**	**5.27**	**.0236**	**6.93**	**.0097**	**7.27**	**.0081**	**0.00**	**.9885**	**0.21**	**.6473**	**0.35**	**.5544**
	
	**Treatment by age**	0.12	.7342	6.55	.0118	2.06	.1543	0.06	.8090	1.55	.2159	1.38	.2418

**10**	**Treatment**	**72.09**	**<.0001**	**68.52**	**<.0001**	**150.65**	**<.0001**	**32.26**	**<.0001**	**108.63**	**<.0001**	**115.45**	**<.0001**
	
	**Age**	3.26	.0736	11.15	.0011	7.22	.0083	0.23	.6300	0.60	.4393	0.64	.4271
	
	**Treatment by age**	**0.83**	**.3636**	**0.04**	**.8433**	**0.05**	**.8172**	**1.00**	**.3186**	**0.20**	**.6556**	**0.05**	**.8202**

**12**	**Treatment**	34.82	<.0001	44.84	<.0001	100.99	<.0001	30.71	<.0001	74.62	<.0001	81.57	<.0001
	
	**Age**	**5.34**	**.0227**	**9.44**	**.0027**	**12.67**	**.0006**	**3.97**	**.0487**	**0.13**	**.7163**	**0.06**	**.8065**
	
	**Treatment by age**	0.42	.5170	0.06	.8007	0.00	.9549	1.06	.3058	0.27	.6020	0.13	.7158

**13**	**Treatment**	**8.29**	**.0048**	**34.69**	**<.0001**	**38.63**	**<.0001**	**10.26**	**.0018**	**67.42**	**<.0001**	**68.29**	**<.0001**
	
	**Age**	5.56	.0201	7.40	.0076	9.48	.0026	0.34	.5637	0.03	.8639	0.09	.7587
	
	**Treatment by age**	**0.01**	**.9293**	**0.45**	**.5018**	**0.45**	**.5051**	**2.15**	**.1458**	**0.02**	**.8820**	**0.02**	**.8913**

LDX: lisdexamfetamine dimesylate; PERMP: Permanent Product Measure of Performance; PERMP-A: PERMP-Attempted; PERMP-C: PERMP-Correct; SKAMP: Swanson, Kotkin, Agler, M-Flynn, and Pelham; SKAMP-A: SKAMP-Attention; SKAMP-D: SKAMP-Deportment.

*Degrees of freedom (df) = 110 for all analyses except the 7.5-hour postdose time point where df = 109 for all analyses.

**Figure 4:**
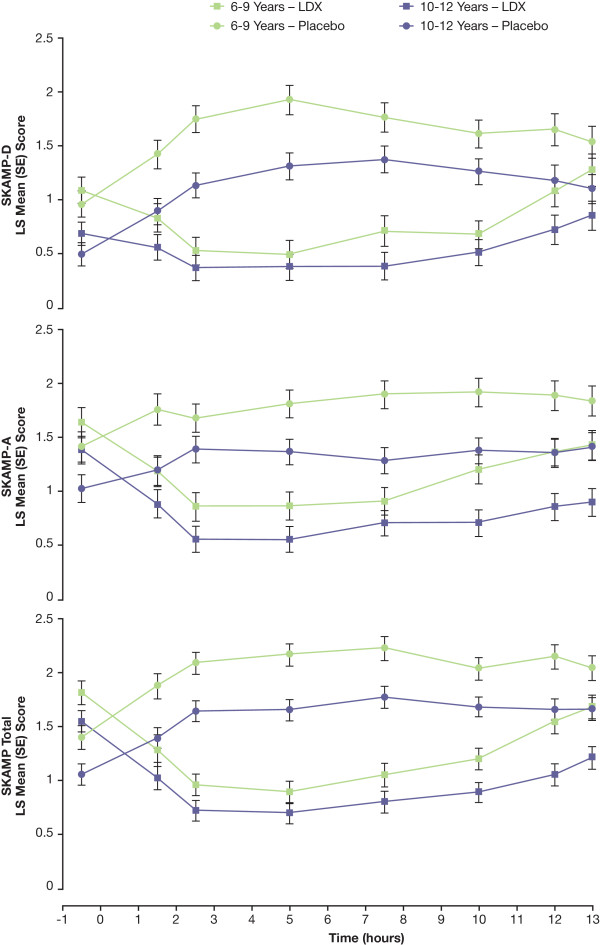
**Postdose LS Mean (SE) SKAMP-D, SKAMP-A, and Total Scores by Time and Age**. Lower SKAMP scores indicate improvement.

**Figure 5:**
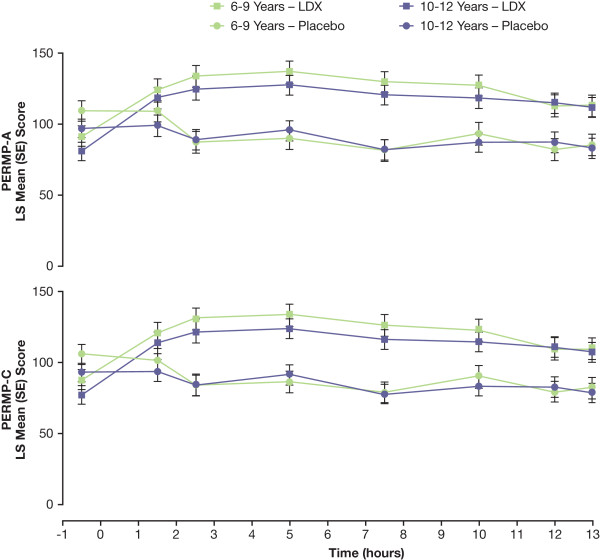
**Postdose LS Mean (SE) PERMP-A and PERMP-C Scores by Time and Age**. Higher PERMP subscale scores are indicative of improvement.

Results of efficacy analyses for postdose time points by age are shown in Figures 4 and 5 and mixed model statistical analysis in Table 3. There were significant effects of age at all time points for SKAMP-D subscale scores, except at the 10-hour time point, with participants aged 10 to 12 years showing less impairment than did those aged 6 to 9 years overall. Significant treatment-by-age interactions were seen at the 2.5- and 5-hour time points.

Results of age analysis for SKAMP total scores were similar to those of SKAMP-D and showed significant effects at all postdose time points, with participants aged 10 to 12 years demonstrating significantly less impairment than did those aged 6 to 9 years overall and a significant treatment-by-age interaction at 5 hours. For SKAMP-A, significant effects of age were seen at all postdose time points, except at 2.5 hours, and the only significant treatment-by-age interaction was observed at the 7.5-hour time point. With LDX treatment, LS mean SKAMP scores for participants aged 10 to 12 years were lower than were those for participants aged 6 to 9 years for all measures at all postdose time points with the exception of the SKAMP quality of work at the 7.5-hour time point. Similarly, LS mean SKAMP scores for participants aged 10 to 12 years were lower than those for participants aged 6 to 9 years for all measures at all postdose time points when receiving placebo, with the exception of the SKAMP quality of work subscale at the 1.5-, 10-, and 13-hour postdose time points. No significant effects for age were noted in PERMP-A and PERMP-C analyses. Significant treatment-by-age interactions were noted at the 5-hour time point for both PERMP-A and PERMP-C measures.

#### Effect size

The predose LS mean (SE) effect size for the SKAMP-D subscale was 0.26 (0.13). Based on effect sizes, LDX demonstrated significant improvement on the SKAMP-D (*P *< .05) compared with placebo from 1.5 hours, the first postdose time point measured, to 13 hours postdose, the last time point measured. The LS mean (SE) treatment effect size over the classroom day on the SKAMP-D was -1.73 (0.18). The magnitude of effect size of LDX treatment as measured by the SKAMP-D subscale was mostly medium to large except for the last time point (13 hours postdose) at which a small to medium effect was observed (Table 4).

**Table 4:** LS Mean (SE) Postdose SKAMP and PERMP Effect Sizes*

Hour	SKAMP-D LS Mean (SE) Effect Size	SKAMP-A LS Mean (SE) Effect Size	SKAMP Quality of Work LS Mean (SE) Effect Size	SKAMP Total LS Mean (SE) Effect Size	PERMP-A LS Mean (SE) Effect Size	PERMP-C LS Mean (SE) Effect Size
1.5	-0.68 (0.14)	-0.57 (0.14)	-0.23 (0.13)	-0.85 (0.15)	0.59 (0.14)	0.74 (0.14)

2.5	-1.41 (0.16)	-1.20 (0.16)	-1.05 (0.15)	-1.89 (0.18)	1.28 (0.16)	1.34 (0.16)

5.0	-1.44 (0.16)	-1.19 (0.16)	-1.24 (0.16)	-1.90 (0.18)	1.40 (0.16)	1.44 (0.16)

7.5	-1.41 (0.16)	-1.27 (0.16)	-1.17 (0.16)	-1.94 (0.19)	1.53 (0.17)	1.56 (0.17)

10.0	-1.12 (0.15)	-1.11 (0.15)	-0.77 (0.14)	-1.64 (0.17)	1.39 (0.16)	1.44 (0.16)

12.0	-0.78 (0.14)	-0.89 (0.15)	-0.75 (0.14)	-1.35 (0.16)	1.15 (0.15)	1.21 (0.16)

13.0	-0.38 (0.14)	-0.80 (0.14)	-0.44 (0.14)	-0.84 (0.14)	1.10 (0.15)	1.11 (0.15)

Mean^†^	-1.73 (0.18)	-1.54 (0.17)	-1.73 (0.18)	-2.41 (0.21)	1.78 (0.18)	1.83 (0.18)

LS: least squares; PERMP: Permanent Product Measure of Performance; PERMP-A: PERMP-Attempted; PERMP-C: PERMP-Correct; SE: standard error; SKAMP: Swanson, Kotkin, Agler, M-Flynn, and Pelham; SKAMP-A: SKAMP-Attention; SKAMP-D: SKAMP-Deportment.

*Negative SKAMP effect sizes indicate improvement with LDX. Positive PERMP effect sizes indicate improvement with LDX.

^†^Mean SKAMP and PERMP scores across the day were calculated employing the model-based calculations using all measures for all participants at all time points across the day.

The predose LS mean (SE) effect size for the SKAMP-A and SKAMP quality of work subscales and SKAMP total scores were 0.45 (0.14), 1.55 (0.17), and 0.94 (0.15), respectively. Based on effect sizes for SKAMP-A and SKAMP total scores, LDX demonstrated improvement compared with placebo from 1.5 hours to 13 hours postdose (Table 4); for SKAMP quality of work subscale, LDX demonstrated improvement compared with placebo from 2.5 hours to 13 hours postdose (Table 4).

The magnitude of effect size of LDX treatment as measured by SKAMP subscale scores (SKAMP-D, SKAMP-A, and SKAMP quality of work) and SKAMP total score effect sizes demonstrated a medium to large effect size of drug vs placebo at most postdose time points (Table 4).

The predose LS mean (SE) effect size for PERMP-A and PERMP-C scores were -0.79 (0.14) and -0.82 (0.14), respectively. The postdose effect size of LDX on PERMP-A and PERMP-C was large and maintained from 1.5 to 13 hours postdose (Table 4).

The mean raw postdose effect sizes for all optimized LDX dose groups (30, 50, and 70 mg/d) were mostly large for SKAMP-D, SKAMP-A, and SKAMP quality of work subscales, and SKAMP total score (Table 5).

**Table 5:** Postdose SKAMP Mean (SE) Effect Size* by Optimized Dose

LDX Dose Group	SKAMP-D	SKAMP-A	SKAMP Quality of Work	SKAMP Total
30 mg/d (n = 46)	-0.85 (0.23)	-0.67 (0.22)	-0.94 (0.23)	-1.13 (0.24)

50 mg/d (n = 47)	-0.86 (0.22)	-0.80 (0.22)	-1.14 (0.24)	-1.18 (0.24)

70 mg/d (n = 20)	-1.14 (0.36)	-0.98 (0.35)	-0.83 (0.34)	-1.24 (0.37)

LDX: lisdexamfetamine dimesylate; SE: standard error; SKAMP: Swanson, Kotkin, Agler, M-Flynn, and Pelham; SKAMP-A: SKAMP-Attention; SKAMP-D: SKAMP-Deportment.

*Negative effect sizes indicate improvement with LDX.

As previously reported, ADHD-RS-IV total score and ADHD-RS-IV inattention and hyperactivity/impulsivity subscale scores decreased from baseline for all doses of LDX during the dose-optimization phase and improved for all doses of LDX vs placebo (by difference in LS means: all *P *< .0001) during the crossover phase [10]. Large treatment effect sizes were observed. The LS mean (SE) treatment effect size was -1.4 (0.16) for ADHD-RS-IV total score and -1.4 (0.16) for inattention and -1.3 (0.16) for hyperactivity/impulsivity subscale scores.

### Safety

Overall safety data have been published previously [10]. There were no deaths or serious AEs reported during this study. Most TEAEs were mild to moderate in severity. During the dose-optimization phase, 110 participants (85.3%) reported TEAEs; the most common TEAEs reported during this phase included decreased appetite (47.3%), insomnia (27.1%), headache (17.1%), irritability (16.3%), affect lability (10.1%), and upper abdominal pain (15.5%). During the crossover phase, the most common TEAEs reported for participants receiving LDX included decreased appetite (6.1%), headache (5.2%), and insomnia (4.3%). Detailed vital signs and electrocardiographic (ECG) data were presented previously [10]. During the dose-optimization phase, there were small increases in systolic blood pressure (SBP), diastolic blood pressure (DBP), and pulse, but no dose-related changes were noted. During the crossover phase, small mean increases in SBP, DBP, and pulse were seen for participants while taking LDX and placebo. No clinically concerning trends in ECG parameters were identified.

The overall incidence of TEAEs in each sex was similar during the dose-optimization phase (Table 6). TEAEs with ≥2% difference between sexes in the dose-optimization phase included upper abdominal pain (males, 16.3%; females, 12.9%), nausea (males, 7.1%; females, 12.9%), decreased weight (males, 2.0%; females, 6.5%), headache (males, 18.4%; females, 12.9%), affect lability (males, 11.2%; females, 6.5%), initial insomnia (males, 6.1%; females, 0.0%), and insomnia (males, 30.6%; females, 16.1%). During the crossover phase (Table 7), when receiving LDX, males had a numerically greater rate of TEAEs (males, 34.5%; females, 28.6%). Differences (≥2%) in rates of TEAEs between sexes when receiving LDX in the crossover phase were upper abdominal pain (males, 2.3%; females, 0.0%), nausea (males, 1.1%; females, 3.6%), headache (males, 4.6%; females, 7.1%), and insomnia (males, 5.7%; females, 0.0%).

**Table 6:** TEAEs by sex in the dose-optimization phase while receiving LDX with an incidence of ≥10% in the dose-optimization and/or crossover phase

AE-Preferred Term	Dose-Optimization Phase	
	Males	Females
	(n = 98)	(n = 31)
	n (%)	n (%)

Any AE	83 (84.7)	27 (87.1)

Abdominal pain upper	16 (16.3)	4 (12.9)

Affect lability	11 (11.2)	2 (6.5)

Decreased appetite	47 (48.0)	14 (45.2)

Headache	18 (18.4)	4 (12.9)

Insomnia	30 (30.6)	5 (16.1)

Irritability	16 (16.3)	5 (16.1)

Nausea	7 (7.1)	4 (12.9)

For tables 6-9: TEAEs were assigned to either the open-label dose-optimization phase or the double-blind crossover phase of the study and were summarized separately. TEAEs that continued uninterrupted from the dose-optimization to the crossover phase without a change in severity were counted only in the dose-optimization phase category. TEAEs with a change in severity across phases or that resolved and then restarted in the crossover phase were counted both in the dose-optimization and crossover arms. TEAEs for which a missing or incomplete start date made it impossible to determine in which phase of the study they started were counted as starting in the dose-optimization phase.

**Table 7:** TEAEs by sex in the crossover phase while receiving LDX with an incidence of ≥10% in the dose-optimization and/or crossover phase

AE-Preferred Term	Crossover Phase	
	Males	Females
	(n = 87)	(n = 28)
	n (%)	n (%)

Any AE	30 (34.5)	8 (28.6)

Abdominal pain upper	2 (2.3)	0 (0.0)

Affect lability	0 (0.0)	0 (0.0)

Decreased appetite	5 (5.7)	2 (7.1)

Headache	4 (4.6)	2 (7.1)

Insomnia	5 (5.7)	0 (0.0)

Irritability	1 (1.1)	0 (0.0)

Nausea	1 (1.1)	1 (3.6)

Male participants receiving LDX had mean (SD) changes in weight from baseline of -1.5 (6.8), -2.0 (6.4), -2.6 (5.7), -2.1 (7.8), and -2.7 (6.2) lb at weeks 1, 2, 3, 4, and 5/6, respectively. Female participants receiving LDX had mean (SD) changes in weight from baseline of -1.9 (2.5), -2.3 (1.9), -3.4 (2.0), -3.5 (2.5), and -2.1 (7.0) lb at weeks 1, 2, 3, 4, and 5/6, respectively.

The overall incidence of TEAEs in each age group was similar during dose-optimization and crossover phases (Tables 8 and 9). In the dose-optimization phase, the incidence rates by dose group at the time of first occurrence of anorexia in the younger group were 10.4%, 0.0%, and 0.0% and in the older group were 1.2%, 0.0%, and 6.3% for participants receiving 30, 50, and 70 mg/d LDX, respectively. The incidence rates by dose group at the time of first occurrence of decreased appetite were 37.5%, 17.4%, and 14.3% in the younger group and 42.0%, 7.8%, and 0.0% in the older group, for participants receiving 30, 50, and 70 mg/d LDX, respectively. In the dose-optimization phase, the incidence rates by dose group at the time of first occurrence of insomnia were 31.3%, 13.0%, and 0.0% in the younger group and 17.3%, 7.8%, and 0.0% in the older group, for participants receiving 30, 50, and 70 mg/d LDX, respectively.

**Table 8:** TEAEs by age group in the dose-optimization phase while receiving LDX with an incidence of ≥10% in the dose-optimization and/or crossover phase

AE-Preferred Term	Dose-Optimization Phase	
	6 to 9 year olds	10 to 12 year olds
	(n = 48)	(n = 81)
	n (%)	n (%)

Any AE	43 (89.6)	67 (82.7)

Abdominal pain upper	8 (16.7)	12 (14.8)

Affect lability	7 (14.6)	6 (7.4)

Anorexia	5 (10.4)	2 (2.5)

Decreased appetite	23 (47.9)	38 (46.9)

Headache	9 (18.8)	13 (16.0)

Insomnia	17 (35.4)	18 (22.2)

Irritability	7 (14.6)	14 (17.3)

**Table 9:** TEAEs by age group in the crossover phase while receiving LDX with an incidence of ≥10% in the dose-optimization and/or crossover phase

AE-Preferred Term	Crossover Phase	
	6 to 9 year olds	10 to 12 year olds
	(n = 42)	(n = 73)
	n (%)	n (%)

Any AE	16 (38.1)	22 (30.1)

Abdominal pain upper	1 (2.4)	1 (1.4)

Affect lability	0 (0.0)	0 (0.0)

Anorexia	0 (0.0)	0 (0.0)

Decreased appetite	2 (4.8)	5 (6.8)

Headache	2 (4.8)	4 (5.5)

Insomnia	3 (7.1)	2 (2.7)

Irritability	1 (2.4)	0 (0.0)

Participants aged 6 to 9 years receiving LDX had mean changes in weight from baseline of -2.5 (6.8), -2.3 (8.1), -3.4 (7.2), -2.2 (9.8), and -2.9 (8.8) lb at weeks 1, 2, 3, 4, and 5/6, respectively. At week 5/6, the mean (SD) weight loss was -0.7 (6.5), -6.6 (11.8), and -1.4 (1.5) lb for younger participants receiving 30, 50, 70 mg/d LDX, respectively. Participants aged 10 to 12 years receiving LDX had mean (SD) changes in weight from baseline of -0.8 (5.1), -1.9 (2.0), -2.3 (2.1), -2.6 (2.5), and -2.3 (3.0) lb at weeks 1, 2, 3, 4, and 5/6, respectively. At week 5/6, the mean (SD) weight loss of participants was -1.7 (3.2), -2.3 (2.4), and -3.1 (3.8) lb for older participants receiving 30, 50, 70 mg/d LDX, respectively.

## Discussion

### Effects of Sex

The effects of sex on treatment response have not been thoroughly studied; however, some sex differences in psychosocial, cognitive, and psychiatric functioning have been noted and may influence response to treatment. Psychosocial functioning in boys and girls is generally similar, but more externalizing behaviors have been identified in boys [16]. Existing data on sex as a moderating factor in treatment response are mixed [11]. In addition to the above clinical data, recent studies in animal models [31] and healthy human adults [32] suggest the potential for sex differences in the physiologic response to agents that interact with the dopaminergic system in the brain. The estrous cycle in female rats modulates amphetamine-stimulated dopamine release and other dopaminergic functions in the striatum [31]. Along similar lines, using brain imaging techniques, Munro et al demonstrated that males had greater amphetamine-stimulated dopamine release in the ventral striatum, anterior putamen, as well as the anterior and posterior caudate nuclei [32]. Additionally, subjective positive effects of amphetamine were greater in men than in women [32]. These preliminary findings are interesting; however, additional clinical and nonclinical studies will be necessary to determine whether these differences in brain physiology will result in demonstrable differences in clinical response.

In the current study, significant effects of sex were noted for SKAMP-D and SKAMP total scores. These effects were not noted for SKAMP-A, SKAMP quality of work, PERMP-A, or PERMP-C. Significant treatment-by-sex interactions were not seen at the majority of time points. Females in both placebo and treatment conditions had lower (ie, improved) scores on all SKAMP total and subscale scores than did males at all time points. While receiving placebo, females had higher (less impaired) scores on the PERMP-A and PERMP-C than did males receiving placebo. With LDX treatment, females and males improved and had similar scores on PERMP-A and PERMP-C. The lack of consistent treatment-by-sex interactions indicated that both males and females responded well to LDX. Further, the results of this analysis support the efficacy of LDX from 1.5 hours to 13 hours postdose in both male and female participants. The duration of effect of LDX does not seem to differ between males and females. Although there appeared to be greater separation between the LDX and placebo conditions among males, females generally showed less impairment prior and subsequent to dosing than did males in the same treatment condition. The beneficial effects of LDX observed may have been restricted by the limited impairment seen in females overall (ie, a floor effect). This finding is similar to that in the COMACS study [14], an analog classroom study of male and female children that used typical laboratory school measures to assess once-daily methylphenidate vs placebo but that likewise was not powered statistically to specifically measure student sex-based differences in stimulant treatment.

However, the results of the post hoc treatment-by-sex analysis presented here differs from the COMACS study, a trial with a similar double-blind, analog classroom design and a similar participant age profile, in which efficacy at early vs later time points differed between males and females [14]. Additionally, statistical analysis of SKAMP scores in the placebo cohort showed differences at predose and most postdose time points between males and females [14]. Interestingly, differences between sexes were not apparent when assessing PERMP scores in the COMACS study [14]. While no statistical analysis was performed here to assess differences between sexes for the placebo cohort alone, the current data similarly appear to show differential characteristics between sexes in the placebo cohort for SKAMP but not PERMP measures. While these sex differences are intriguing, any consideration of cause would be speculative in nature. These findings indicate a need for continued close examination of differential presentation of ADHD symptoms and symptom clusters in females and males.

Although the distribution of participants by sex and racial group was similar, there were some differences between the current study and the COMACS study in participant demographic characteristics. Mean age was slightly higher in the current study than in the COMACS study (10.1 vs 9.58 years), and all participants in the current study had combined-type ADHD, whereas approximately 82% in the COMACS study had combined-type ADHD [14]. Although the application of different entry criteria (ADHD-RS-IV scores ≥28 vs no severity cutoff) and baseline ADHD assessment measures (ADHD-RS-IV vs Swanson, Nolan and Pelham scale [SNAP]) in the current study and the COMACS study, respectively, make it difficult to specifically assess similarity in baseline ADHD symptom severity [14], overall SNAP baseline inattention and overactivity/impulsivity scores between 1.09 and 1.28 suggest that ADHD severity in the COMACS study population may have been somewhat less than the severity in the current study (mean ADHD-RS-IV baseline score was 42.4).

### Effects of age

Significant effects for age were noted in SKAMP-D, SKAMP-A, and SKAMP total scores, but not for PERMP-A or PERMP-C measures. Significant treatment-by-age interactions were not seen at the majority of time points for both SKAMP and PERMP measures. Both age groups responded to treatment. Older children in both the placebo and the treatment condition groups had lower SKAMP total and subscale scores than did younger children at predose and in a majority of the measured postdose time points. These findings of lower scores suggesting less impairment in older than younger children are in general agreement with previous findings that highlight age-dependent decline of symptoms and age-dependent changes in the functional expression of symptoms of ADHD [33-35]. The duration of effect of LDX does not seem to differ between age groups.

### Effect size

From 1.5 to 13 hours postdose, the effect size of LDX for the primary efficacy variable, SKAMP-D, was medium to large at most postdose time points, as was the mean effect size over the course of the day. It should be noted that overall mean effect sizes across the day for SKAMP-D and the other SKAMP and PERMP measures were calculated in the model-based system and included all appropriate assessment values for all time points, so variance of these calculated means is commensurately small. The mean overall raw effect sizes for all optimized doses of LDX (median optimized LDX dose was 50 mg/d) were medium to large for SKAMP-D, SKAMP-A, SKAMP quality of work, and SKAMP total scores. These results are not indicative of dose response because participants were not randomized to a dose group but rather were clinically titrated to LDX doses studied in the double-blind portion of the study. Thus, in the analog classroom setting, LDX was effective in reducing ADHD symptoms of deportment and attention from 1.5 to 13 hours postdose based on optimized dosing with mostly medium to large effect sizes for a broad array of assessments, including clinician observations, classroom behavior, and test of math performance.

Large treatment effect sizes were also observed for DSM-IV-TR–defined ADHD symptoms as assessed by ADHD-RS-IV total and subscores. These findings are consistent with a previous report that demonstrated similarly large ADHD-RS-IV effect sizes (1.39, 1.42, and 1.73 for 30, 50, and 70 mg/d LDX, respectively) in children with ADHD receiving LDX in a clinical trial with a longitudinal design [36].

Meta-analyses of multiple studies have concluded that effect sizes for the treatment of ADHD average 0.91 to 0.95 for stimulant agents [24,37]. A randomized, double-blind, placebo-controlled study of the MPH-ER formulation (20, 40, or 60 mg/d) and OROS-MPH formulation (18, 36, or 54 mg/d) in the treatment of ADHD in children (6-12 years of age) examined effect sizes at various time points. Dosing was based on preexisting methylphenidate dosing. Effect sizes for MPH-ER as measured by SKAMP-D ranged from 0.06 (at 12 hours postdose) to 0.89 (at 3 hours postdose). Effect sizes for OROS-MPH ranged from 0.25 (at 12 hours postdose) to 0.66 (at 6 hours postdose) [6]. A placebo-controlled, double-blind, multiple-crossover study of methylphenidate (approximately 0.3 mg/kg/d) that studied the same participants as children and as adolescents in a summer treatment program reported an overall effect size, the product of 12 different measures (including IOWA Conners Rating Scale-counselor and IOWA Conners Rating Scale-teacher ratings, assessment of classwork completed and correct, and behavioral assessments of rule violations and other negative and positive behaviors), of 0.82 in children and 0.59 in adolescents [38].

Small to medium effect sizes favoring placebo were observed at predose time points for SKAMP-A and SKAMP-D, and large effect sizes at predose favoring placebo were observed for SKAMP quality of work, SKAMP total, PERMP-A, and PERMP-C scales. Although many factors may have contributed to this observation, residual drug from the previous treatment day may have played a role in this finding as has been seen in another study with an amphetamine-based long-acting stimulant [39]. Pharmacokinetic data showing residual plasma levels were measurable prior to dosing with long-acting stimulants [40,41]. The impact of these pretreatment differences on the ultimate relationship between postdose LDX and placebo responses is unclear.

Overall incidence of TEAEs in this study has been described in detail [10] and is generally similar to that reported in other pediatric studies of LDX [8] and other long-acting stimulants [9,42,43] where active treatment was begun after a washout period for prior treatments. A consistent pattern of greater incidence of TEAEs in either age group did not emerge. The incidence of anorexia was higher in the younger group than in the older group. Although the incidence of decreased appetite was roughly equivalent, the greater incidence of weight decrease as a reported AE in participants aged 6 to 9 years may be related in part to the higher incidence of anorexia in this age group. The incidence of insomnia was 35.4% in the younger group and 22.2% in the older group. When assessed by sex, overall incidence of TEAEs was similar between boys and girls. For some commonly reported TEAEs with LDX treatment (eg, insomnia, headache, and affect lability), incidence was somewhat higher in boys than in girls.

### Limitations

The current analysis has some limitations. As these are post hoc analyses and not part of the a priori planned analyses, they were not adequately powered to make direct comparisons of subgroup differences in efficacy variables or AE incidence. The relatively small group sizes and the approximately 3:1 ratio of boys to girls in the study make it difficult to form firm conclusions regarding possible sex effects. It should be noted, however, that this sex disparity is improved from previous trials that have shown male-to-female ratios as high as 9:1 [17,44-46]. This improvement may be attributable to better identification and diagnosis of female individuals with ADHD.

While the TEAE tables may appear to show fewer TEAEs during the crossover phase than in the preceding dose-optimization phase, one should consider the relative duration of treatment exposure during the 2 phases, as well as the manner in which TEAEs were reported. TEAE incidence rates are based on newly occurring events at each weekly assessment during the dose-optimization and crossover phases. As such, continuing TEAEs would only appear at the assessment in which they were first reported. Similarly, when considering the relative incidence of TEAEs during the dose-optimization phase, readers should remember that TEAEs reported by dose may be related to timing of dose escalation during the optimization process rather than to dose-dependent effects. Thus, those TEAEs occurring during the first week of dose-optimization treatment would be reported for 30 mg/d LDX since all participants began dosing at 30 mg/d LDX; those TEAEs first occurring during the second week would be ascribed to either 30 mg/d or 50 mg/d LDX, and so on. Based on the available data from this study, dose dependence of TEAEs cannot be assessed.

## Conclusions

Although the study was not adequately powered to make direct comparisons of subgroups, the results of this analysis support the efficacy of LDX from 1.5 hours to 13 hours postdose in both boys and girls with medium to large mean effect sizes across the day.

## Abbreviations

ADHD: attention-deficit/hyperactivity disorder; ADHD-RS-IV: ADHD Rating Scale IV; AE: adverse event; COMACS: Comparison of Methylphenidates in an Analog Classroom Setting; DBP: diastolic blood pressure; DF: degrees of freedom; DSM-IV-TR: *Diagnostic and Statistical Manual of Mental Disorders, Fourth Edition, Text Revision*; ECG: electrocardiogram; ITT: intention-to-treat; LDX: lisdexamfetamine dimesylate; LS: least squares; MedDRA: *Medical Dictionary for Regulatory Activities*; MTA: Multimodal Treatment Study of Children With ADHD; MPH-ER: methylphenidate extended-release; OROS-MPH: osmotic-release oral system methylphenidate; PATS: Preschool ADHD Treatment Study; PERMP: Permanent Product Measure of Performance; PERMP-A: PERMP number attempted; PERMP-C: PERMP number correct; SBP: systolic blood pressure; SD: standard deviation; SE: standard error; SKAMP: Swanson, Kotkin, Agler, M-Flynn, and Pelham; SKAMP-A: SKAMP-Attention; SKAMP-D: SKAMP-Deportment; SNAP: Swanson, Nolan and Pelham scale; SWMD: standardized weighted mean difference; TEAE: treatment-emergent adverse event.

## Competing interests

**SW**, PhD, is a consultant for Abbott, Eli Lilly and Company, McNeil Consumer & Specialty Pharmaceuticals, Next Wave Pharmaceutical, NIMH, NuTec, Shire US Inc., and Taisho; has received research support from Addrenex, Eli Lilly and Company, McNeil, New River, NICHD, NIMH, Novartis, Otsuka, Psychogenics, Quintiles, Shionogi, and Shire US Inc.; and is a speaker for McNeil Consumer & Specialty Pharmaceuticals and Shire US Inc; **SHK**, PhD, has received research support and/or consultant fees from Addrenex Pharmaceuticals Inc, CoMentis Inc, the Environmental Protection Agency, the National Institute on Drug Abuse, the National Institute of Environmental Health Sciences, the National Institute of Mental Health, the National Institute of Neurological Disorders and Strokes, Otsuka Pharmaceuticals, Shire Laboratories Inc., and Supernus Pharmaceuticals; **ACC**, MD, is a consultant for Novartis and Shire; is a speaker for Bristol-Myers Squibb, GlaxoSmithKline, Novartis, and Shire; has received research support from Abbott, Bristol-Myers Squibb, Johnson & Johnson Pharmaceutical Research & Development, LLC, Lilly USA, LLC, NextWave, Novartis, Ortho-McNeil-Janssen Scientific Affairs, Shire, and Somerset; **BA**, MS, is an employee of Shire and holds stock and/or stock options from Shire.

## Authors' contributions

**SW **was an investigator on the parent study and participated in data acquisition, analysis, interpretation, and presentation. SW was fully involved in drafting the manuscript and revising the intellectual content of this manuscript. She has given final approval of this version; **SHK **was an investigator on the parent study and participated in data acquisition, analysis, interpretation, and presentation. SHK was fully involved in drafting the manuscript and revising the intellectual content of this manuscript. He has given final approval of this version; **ACC **was an investigator on the parent study and participated in data acquisition, analysis, interpretation, and presentation. ACC was fully involved in drafting the manuscript and revising the intellectual content of this manuscript. She has given final approval of this version. Statistician **BA **was involved in all post hoc data analysis, interpretation, and presentation. He was fully involved in drafting and revising the intellectual content of this manuscript. BA has given final approval to this version.
